# Supplementary material for: Engineered mRNA-expressed antibodies prevent respiratory syncytial virus infection
Source: Nat Commun. 2018 Oct 1;9:3999. doi: 10.1038/s41467-018-06508-3 (PMC6167369; doi:10.1038/s41467-018-06508-3)
Supplement: Supplementary file 1 — Supplementary Information [file 41467_2018_6508_MOESM1_ESM.pdf]

Engineered mRNA-expressed antibodies prevent respiratory syncytial virus infection

Tiwari and Vanover et al.

Supplementary Figures

Supplementary Figure 1 – Contrast adjustments to characterize aPali staining

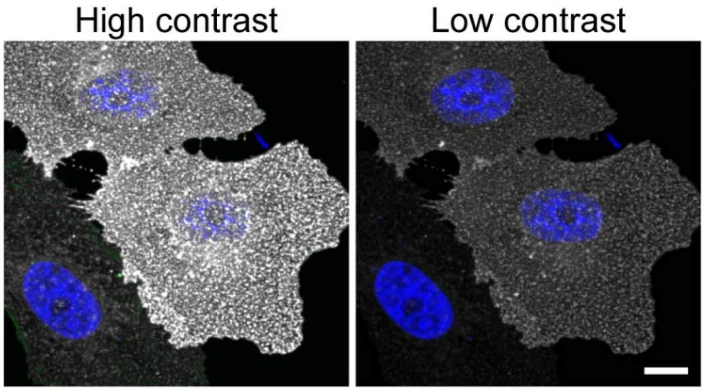

Vero cells were transfected with 1 µg of aPali mRNA. 24 h later, cells were fixed, permeabilized, and stained with a donkey anti-human secondary antibody (white). Images are displayed with high contrast (lower white point) or low contrast (higher white point). Scale bar represents 10 µm.

Supplementary Figure 2 – aPali and sPali prevent RSV infection in transfected A549 cells

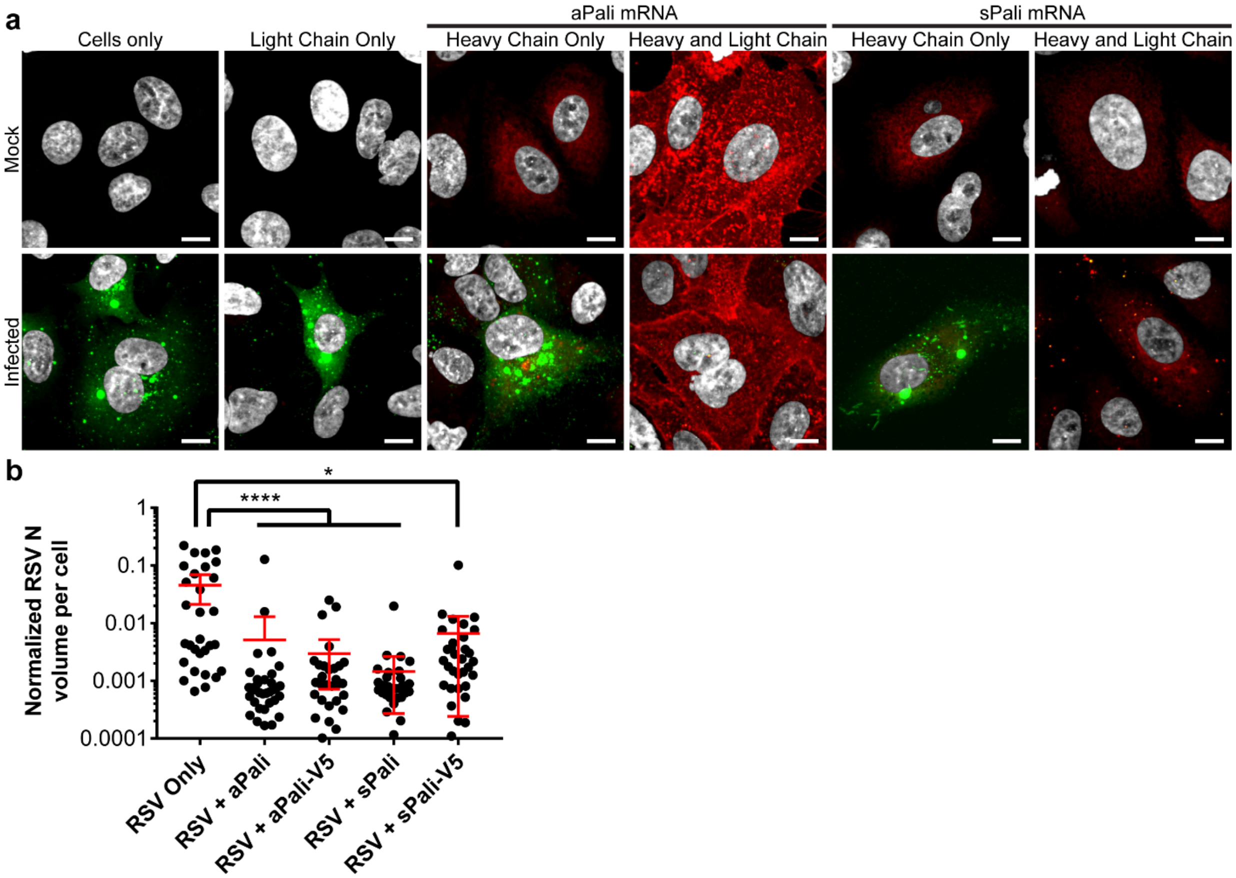

(a) A549 cells were transfected with vehicle control or 1 µg of either light chain only, aPali heavy chain only, aPali heavy chain and light chain, sPali heavy chain only, or sPali heavy and light chain mRNAs. After overnight incubation, cells were infected or mock infected for 24 h before being fixed and stained for RSV N (green) and aPali or sPali (red). Scale bar represents 10 µm. **(b)** Quantification of the mean volume of the RSV N signal per cell from microscopy images in part **(a)** as well as cells transfected with aPali and sPali heavy chains with a light chain encoding a V5 peptide tag. Error bars represent 95% confidence intervals. Single asterisk indicates $p<0.05$ while multiple asterisks indicate $p<0.0001$ (Kruskal-Wallis with Dunn's multiple comparisons against RSV Only group). Results represent mean of two independent experiments.

Supplementary Figure 3 – aPali reduces RSV titer on transfected Vero cells

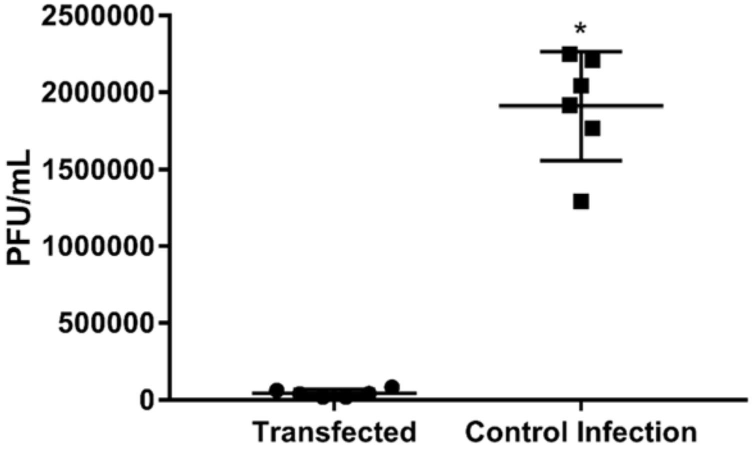

Vero cells were transfected with 1 µg of aPali mRNA or left untransfected. After 24 h, cells were infected with 50 PFU of RSV A2. Virion titer was determined by plaque assay directly on the transfected or control cells. Error bars indicate standard deviation. Asterisk indicates $p<0.05$ (Wilcoxon test). Results represent mean of two independent experiments.

Supplementary Figure 4 – Low aPali mRNA concentration does not result in enhanced infection

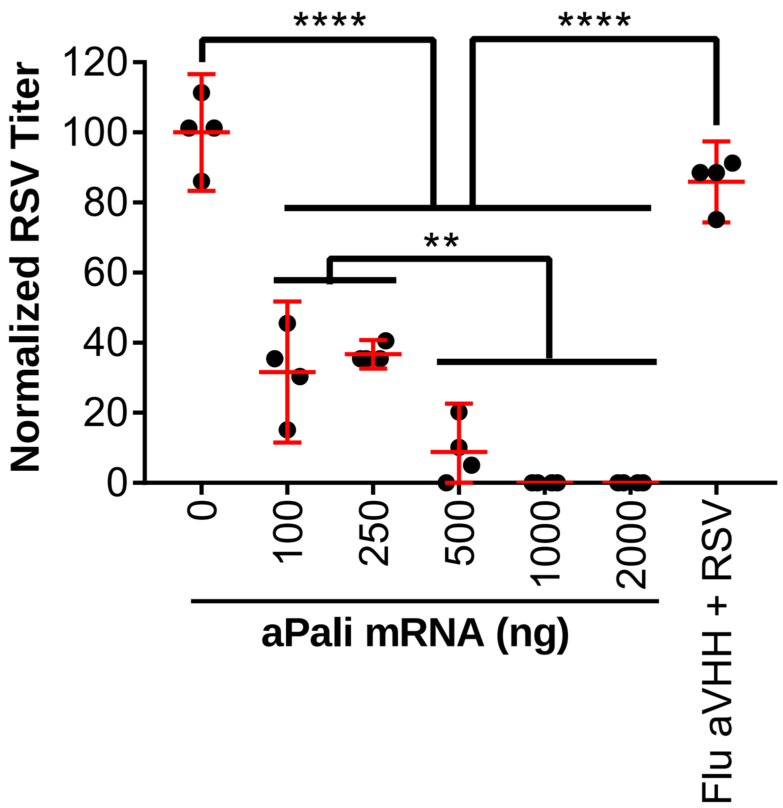

Vero cells were transfected with indicated amount of aPali mRNA, 1 µg Flu aVHH mRNA, or left untransfected. After 24 h, cells were infected with 50 PFU of RSV A2. Virion titer was determined by plaque assay directly on the transfected or control cells. Error bars indicate standard deviation. Two asterisks indicate p<0.005 and four asterisks indicate p<0.0001 (one-way ANOVA with Holm-Sidak multiple comparisons).

Supplementary Figure 5 – dSTORM imaging of untransfected cells infected with RSV

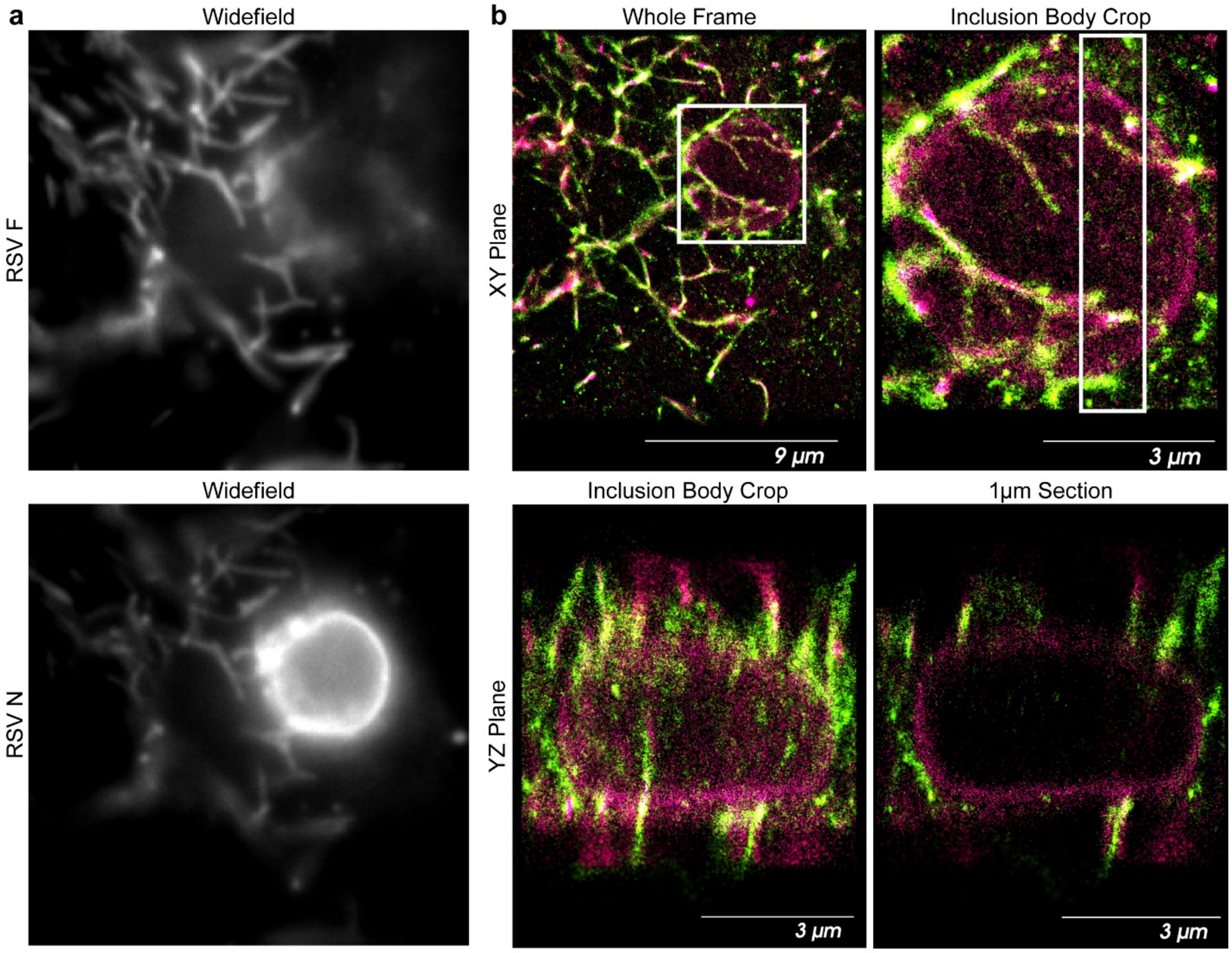

(a) Untransfected Vero cells were infected with RSV at MOI of 1. At 24 hpi, cells were fixed, stained with for RSV F (top) and RSV N (bottom) and imaged using the widefield function on the Vutara. **(b)** The same region from part **(a)** was analyzed using dSTORM, with RSV F pseudocolored green and RSV N pseudocolored magenta. Cropped inclusion body is indicated by the white box in the whole frame view. The section of the inclusion body is indicated by the white rectangle in the cropped image. XY (top) and YZ (bottom) cross-sections are shown.

Supplementary Figure 6 – Labeled aPali mRNA biodistribution in mice lungs

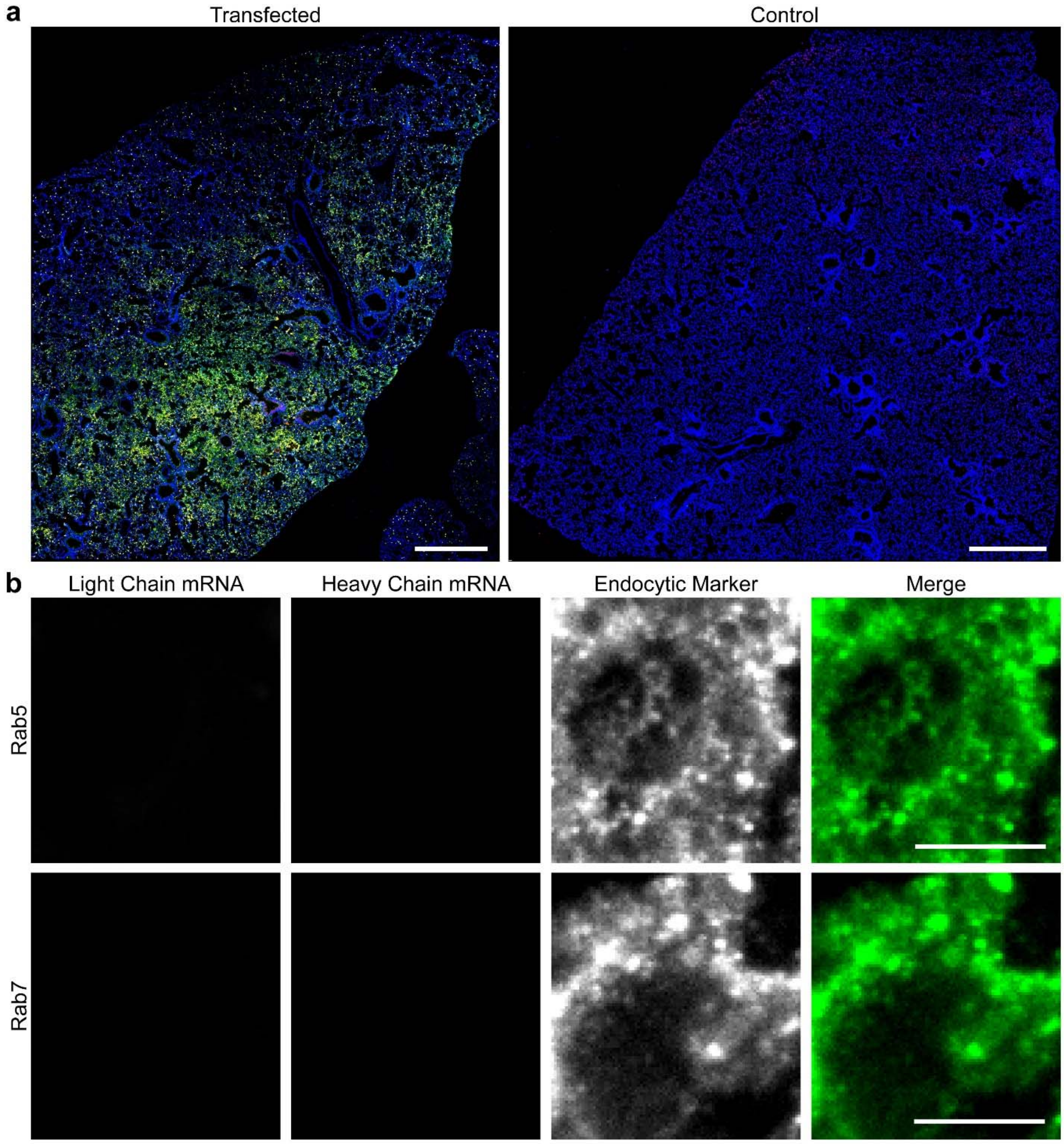

(a) Cy3B labeled light chain (red) and DyLight 650 labeled heavy chain (green) mRNA was transfected into the lungs of mice (left). Saline was injected as a negative control (right). At 4 hours, lungs were excised and cryosectioned before being imaged. Scale bar represents 750 µm. **(b)** Control tissue sections from part **(a)** were stained for Rab5 or Rab7 (green) for early or late endosomes, respectively. Scale bar represents 5 µm.

Supplementary Figure 7 – NearIR labeled commercially available palivizumab biodistribution

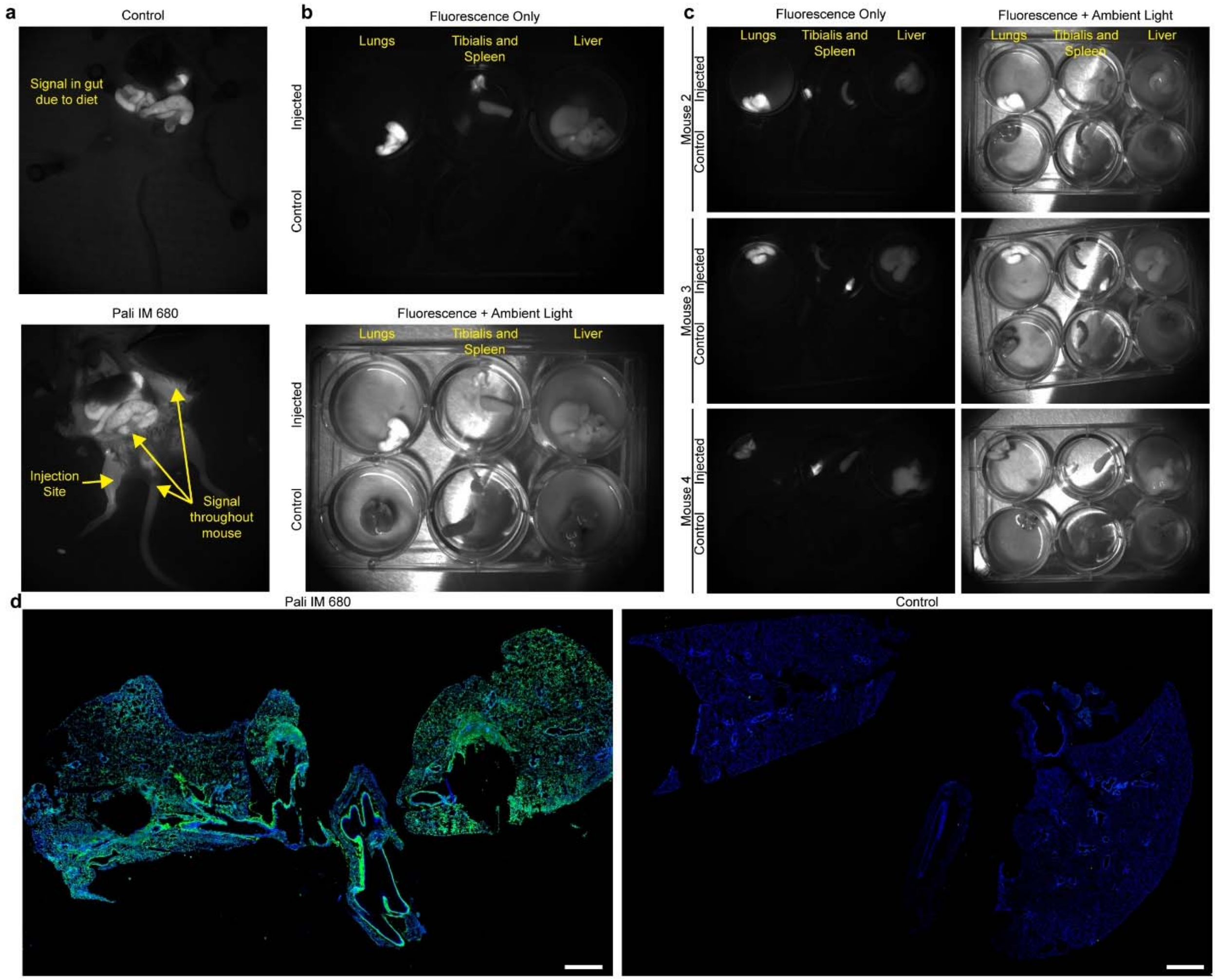

(a) DyLight 680 labeled palivizumab was delivered into the tibialis of mice. Saline was injected as a negative control. After 24 h, mice were sacrificed and dissected. Mice were imaged using the Fluobeam Near-IR imager. In both animals, the gut is fluorescent due to the diet. **(b)** Specific organs from injected animals, including the lungs (left), injected tibialis muscle, spleen (middle), and liver (right) were excised and imaged. Images were taken with the ambient light off (top) and on (bottom) to provide context for the control animals. **(c)** Three other mice were processed and imaged as in part **(b).** **(d)** DyLight 680 labeled palivizumab (green) was delivered into the tibialis of mice (left). Saline was injected as a negative control (right). At 24 hours, lungs were excised and cryosectioned before being imaged. Scale bar represents 750 μm.

Supplementary Figure 8 – C16 does not affect inhibition of RSV by aPali mRNA prophylaxis

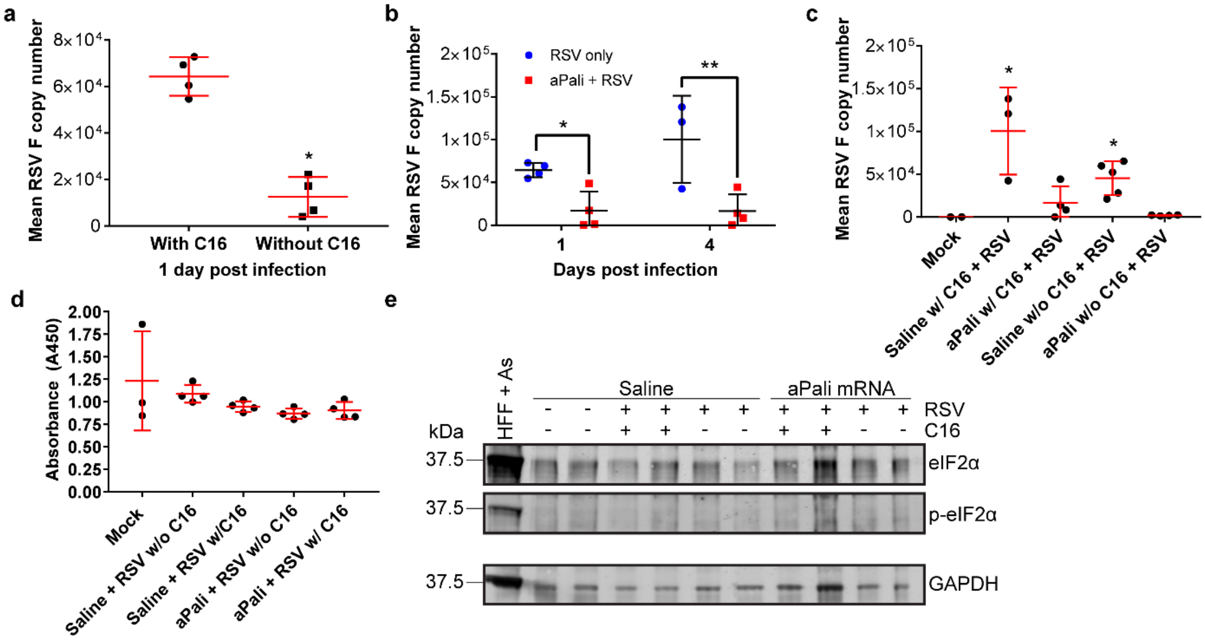

(a) Mice lungs were transfected with saline with or without C16. 24 hours later, mice were infected with RSV L19. At 1 day post infection, mice were sacrificed and lungs were excised and processed for mRNA extraction. Mean RSV F copy number was determined by qRT-PCR. Error bars represent standard deviation. Asterisk indicates $p<0.05$ (Mann-Whitney test). **(b)** Mice lungs were transfected with 100 µg of aPali mRNA. 24 hours later, mice were infected with RSV L19. At 1 and 4 days post infection, mice were sacrificed and lungs were excised and processed for mRNA extraction. Mean RSV F copy number was determined by qRT-PCR. Error bars represent standard deviation. Asterisk indicates $p<0.05$ while multiple asterisks indicates $p<0.005$ (two-way ANOVA with Holm-Sidak multiple comparisons). **(c)** Mice were either transfected with saline or 100 µg of aPali mRNA, either with or without C16. 24 hours later, mice were infected with RSV L19. At 4 dpi, mice were sacrificed and lungs were excised and processed for mRNA extraction. Mean RSV F copy number was determined by qRT-PCR. Error bars represent standard deviation. Asterisk indicates $p<0.05$ (Kruskal Wallis with Dunn's multiple comparisons versus Mock controls). **(d)** The lungs from mice in part **(c)** were processed for protein extraction. Lysates were then assayed by ELISA for phosphorylated eIF2α content. All values were corrected by subtracting mean absorbance of wells containing media only. Center line indicates mean absorbance, while error bars indicate standard deviation. $p>0.05$ by one-way ANOVA. **(e)** Lysates from two mice per group in part **(d)** were assayed by western blot for phosphorylated and total eIF2α on the same blot. Blot displaying GAPDH was used to demonstrate the same tissue processing protocol across samples. Human foreskin fibroblasts (HFF) were treated for 1 h with sodium arsenite (As) as a positive control for PKR activation. 30 µg of protein lysates was loaded in all cases.

Supplementary Figure 9 – Use of *in vivo*-jetPEI results in rapid weight loss in transfected mice

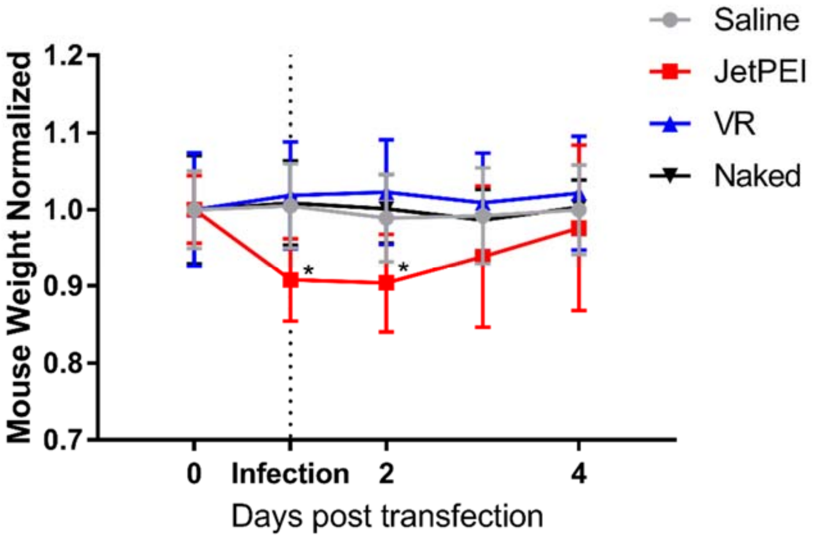

Mice from the experiment in Fig. 4a were weighed each day after transfection. Weights were normalized by the mean of day 0 in each group. Y-axis begin at 0.7 to better show the differences in weight between the treatment groups. Error bars represent 95% confidence interval. Asterisk indicates $p<0.05$ (two-way ANOVA, Tukey's multiple comparisons).

Supplementary Figure 10 – Extent of RSV infection in transfected mice

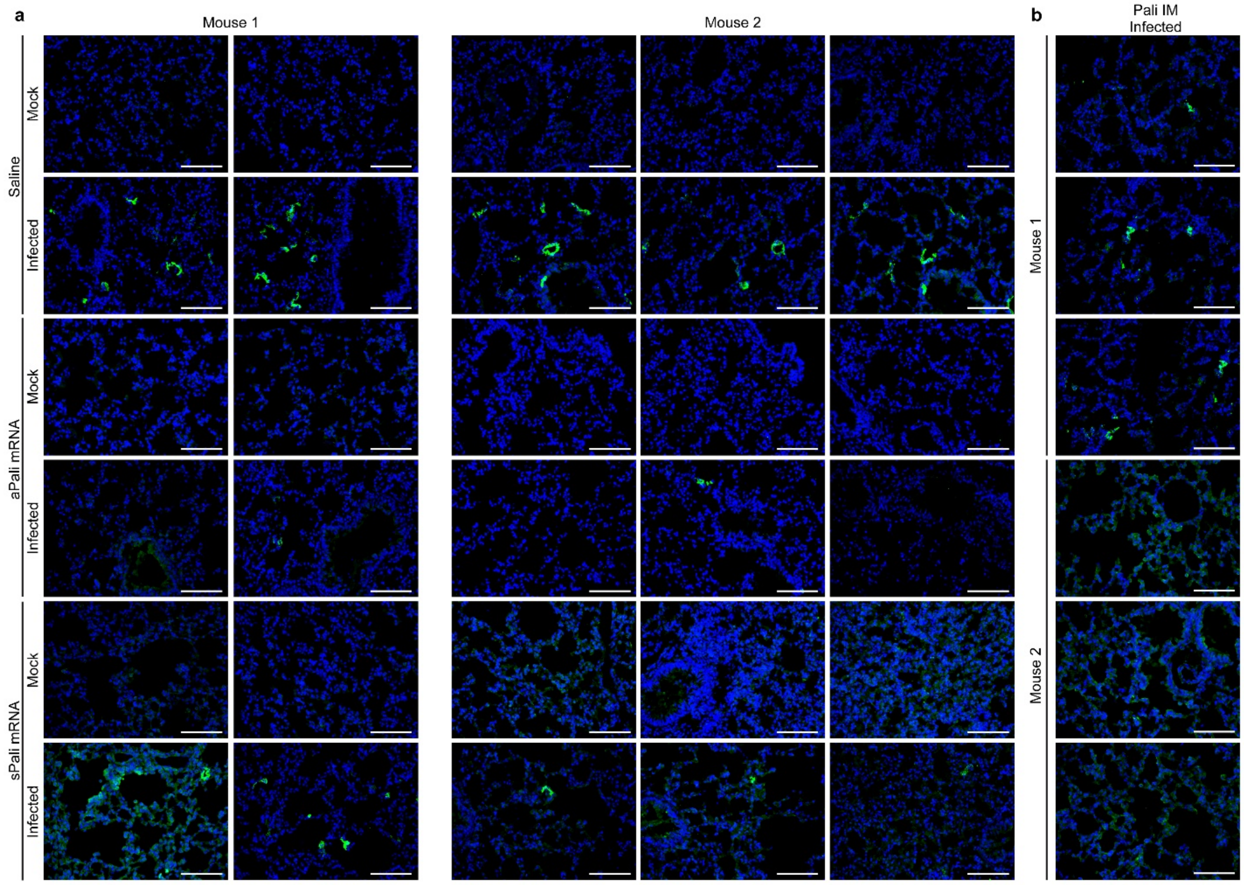

(a) Additional tissue sections were imaged from the experiment in Fig. 5d. Staining for panRSV is in green. Scale bar represents 100 µm. **(b)** Mice were injected IM into the tibialis muscle with 300 µg of palivizumab and infected with RSV L19 the next day. At 4 dpi, animals were sacrificed and lungs were excised, cryosectioned and stained with panRSV (green). Scale bar represents 100 µm.

Supplementary Figure 11 – Cytokine levels in transfected and infected mice.

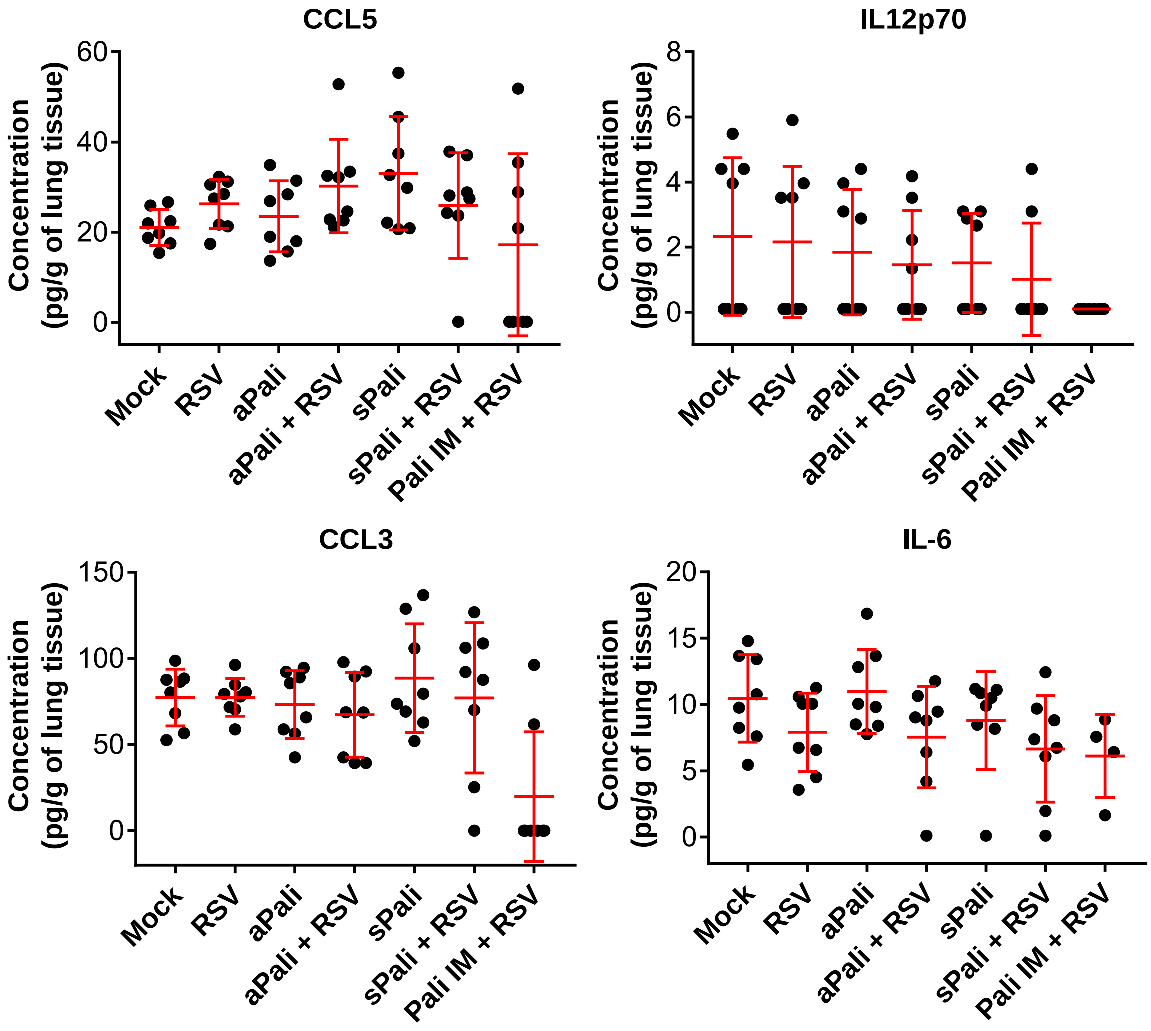

Lung homogenates from the experiment in Fig. 5a and b were analyzed by bead-based immunoassay for cytokine protein levels of CCL5, IL12 p70, CCL3, and IL-6. Error bars represent standard deviation. Undetermined values were set to 0.1 pg/mL. No group was found to be significantly different from another (Kruskal Wallis).

Supplementary Figure 12 – Aerosol delivery of mRNA diluted in water results in no significant cytokine response

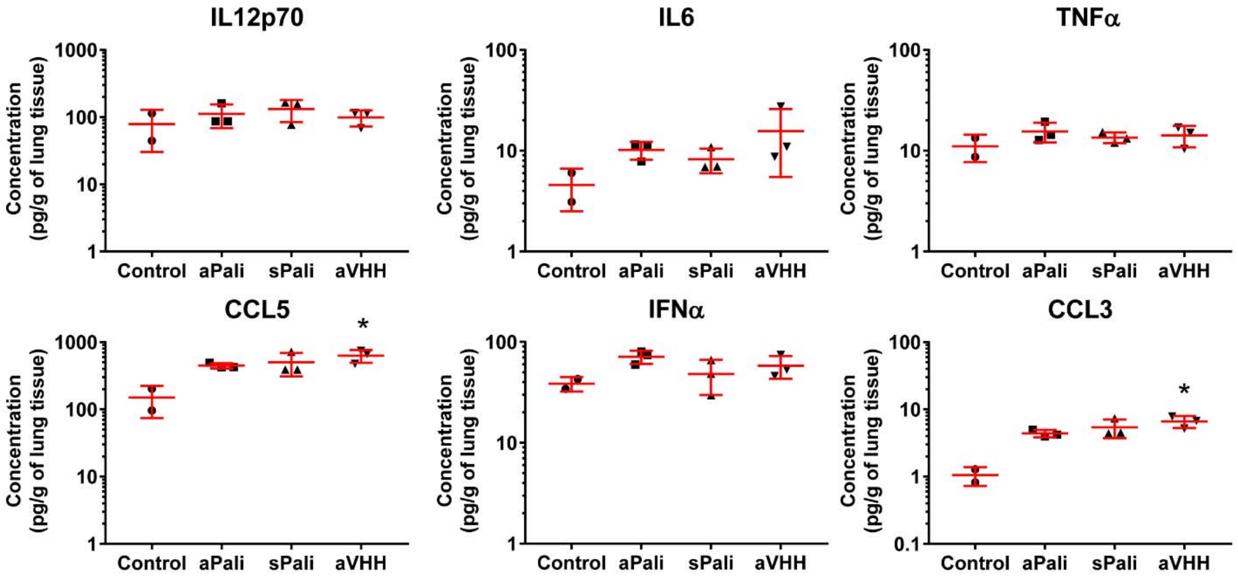

Lung homogenates were analyzed by ELISA for cytokine protein levels of IL12 p70, IL-6, TNFα, CCL5, IFNα, and CCL3. Cytokine concentrations were normalized by lung weight. Error bars represent standard deviation. Asterisk indicates significant difference from control group, $p<0.05$ (Kruskal Wallis with Dunn's multiple comparisons).

Supplementary Figure 13 – RSV aVHH mRNA prevents RSV infection in A549 cells

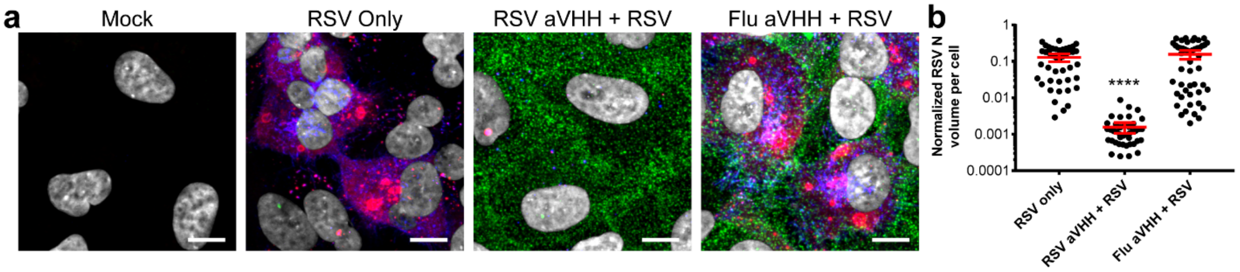

(a) A549 cells were transfected with vehicle control or 1 µg of RSV aVHH or Flu aVHH mRNAs. After overnight incubation, cells were infected or mock infected for 24 h before being fixed and stained for RSV N (green), panRSV (blue), and aVHH (red). Scale bar represents 10 µm. **(b)** Quantification of the mean volume of the RSV N signal per cell from microscopy images in part **(a)**. Error bars represent 95% confidence intervals. Asterisks indicate p<0.0001 (Kruskal-Wallis with Dunn's multiple comparisons).

Supplementary Figure 14 – Transfection with RSV sVHH mRNA prevents RSV infection by plaque assay

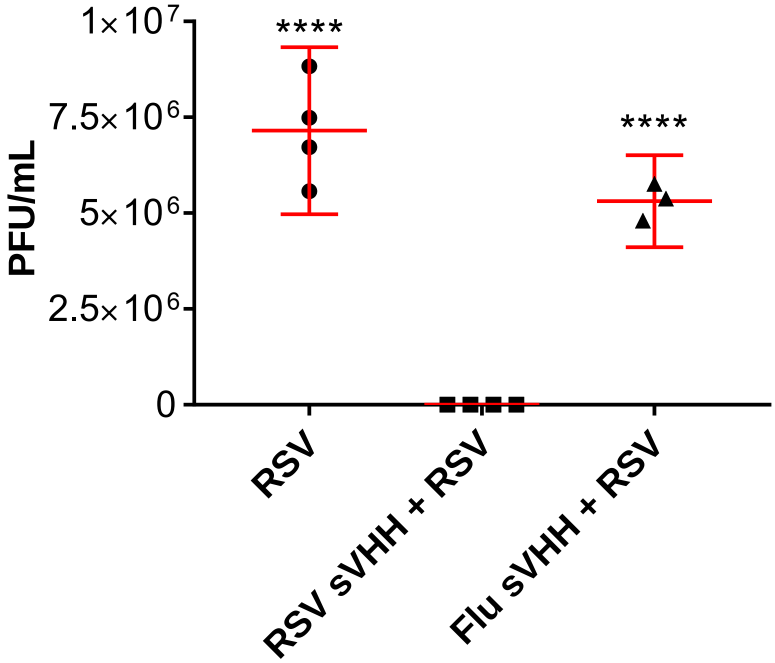

Vero cells were transfected with 1 µg of either RSV sVHH or Flu sVHH mRNA or left untransfected. After 24 h, cells were infected with 50 PFU of RSV A2. Virion titer was determined by plaque assay directly on the transfected or control cells. Error bars indicate 95% confidence intervals. Asterisks indicates $p<0.0001$ (one-way ANOVA with Holm-Sidak multiple comparisons). Results represent mean of two independent experiments.

Supplementary Tables

Supplementary Table 1 – Number of animals used per experiment

Experiments	Groups	No. of animals per group	Total animals per experiment
aPali Expression using microscopy	Control, aPali and sPali	2 each	6
Vehicle test	Control, RSV, Viromer Red, *invivo*Jet-PEI, Naked mRNA	3 each	15
mRNA titration	Control, 20 μg, 100 μg, and Pali IM	3 control, 4 aPali, 5 Pali IM	16
Palivizumab 680	Control and Palivizumab	3 each	6
mRNA distribution in lung	Control and aPali	2 each	4
With and without C16 and different infection length	With and without C16; 1 and 4 dpi; Control	4 for all except 3 for day 4 RSV only	19
Cytokine response to mRNA	Control, aPali, sPali, RSV aVHH	2 control, 3 each	11
aPali expression using Flow Cytometry	Control and aPali	1 control, 3 aPali	4
RSV Challenge	Control, RSV only, aPali, aPali+RSV, sPali, sPali+RSV,	10 (8 for RSV titers and 2 for microscopy)	60
Irrelevant mRNA Controls	aPali HC+RSV and Flu aVHH+RSV	4 each	8
aVHH expression	Control, RSV aVHH, and RSV aVHH+RSV	2 each	6
aVHH Challenge	Control, RSV, RSV aVHH+RSV	3 each	9
Persistence Challenge	Control, RSV, aPali+RSV, sPali+RSV, RSV aVHH+RSV, RSV sVHH+RSV, Flu aVHH+RSV	3 each	21
		**Total**	**185**

Supplementary Table 2 – Primers

Primer 1	Forward Primer for pMA7:VHH at 3' end insertion site	TGATAAGCTGCCTTCTGCGGGGCTTGCCTTC
Primer 2	Reverse Primer for pMA7:VHH at 3' end insertion site	GCACTCGCCCCGGTTGAAGCTCTTGGTCACG
Primer 3	Forward Primer to create insert with V5 tag	CGTGACCAAGAGCTTCAACCGGGGCGAGTGCGGCAAGCCCATCCCCAACCCCCTGCTGGGCCTGGACAGCACCTG
Primer 4	Reverse Primer to create insert with V5 tag	GAAGGCAAGCCCCGCAGAAGGCAGCTTATCAGGTGCTGTCCAGGCCCAGCAGGGGGTTGGGGATGGGCTTGCCGC
Primer 5	Forward Primer to place pMA7 overlaps on 5' end of VHH geneblock	GCGACGATTGGCGGAAGGCCGTCAAGGCCGCATTTTTAAG
Primer 6	Reverse Primer to place pMA7 overlaps on 5' end of VHH geneblock	CGGGCAGTGAGCGGAAGGCCCATGAGGCCCAGTTTTTG
Primer 7	Forward Primer for to amplify linear pMA7 PCR product	GGCCTTCCGCTCACTGCC
Primer 8	Reverse Primer for to amplify linear pMA7 PCR product	GGCCTTCCGCCAATCGTC
Primer 9	RSV F gene (standard) Forward	CTTTCTTCCCACAAGCTGAAAC
Primer 10	RSV F gene (standard) Reverse	GTGGTGGATTTACCAGCATTTAC
Primer 11	RSV F-qPCR Forward*	AACAGATGTAAGCAGCTCCGTTATC
Primer 12	RSV F-qPCR reverse*	CGATTTTTATTGGATGCTGTACATTT
Probe	RSV F probe*	TGCCATAGCATGACACAATGGCTCCT

All sequences are presented in 5’ to 3’ direction. *These sequences are from Mentel et. al. 2003[1].

Supplementary References

1. Mentel, R., Wegner, U., Bruns, R. & Gürtler, L. Real-time PCR to improve the diagnosis of respiratory syncytial virus infection. *Journal of Medical Microbiology* **52,** 893–896 (2003).
